# Untargeted metabolomics and in silico analysis identifies potential DNA gyrase inhibitors from an endophytic fungus *Alternaria alternata* P02PL2

**DOI:** 10.1007/s00253-026-13966-z

**Published:** 2026-07-23

**Authors:** Aviwe N. Matandela, Sphamandla E. Mtambo, Wonder P. Nxumalo, Sizwe I. Ndlovu

**Affiliations:** 1 Department of Biotechnology and Food Technology, Faculty of Science, University of Johannesburg, Johannesburg, South Africa; 2 Rand Water Academy, Rand Water, PO Box 1127, Johannesburg, South Africa; 3 Department of Pharmacology, Faculty of Health Sciences, University of Pretoria, Pretoria, South Africa; 4 NMISA, National Metrology Institute of South Africa CSIR Campus, Building 5, Meiring Naude Road, Brummeria, Pretoria South Africa; 5 Unit for Environmental Sciences and Management, Faculty of Natural and Agricultural Sciences, North-West University, Potchefstroom, 2520 South Africa

**Keywords:** Antimicrobial resistance, Endophytic fungi, OSMAC approach, Secondary metabolites, DNA gyrase inhibitors, Virtual screening

## Abstract

**Abstract:**

The escalating threat of multidrug-resistant (MDR) bacterial pathogens underscores the urgent need for novel antimicrobial agents. Here, we combined a one-strain many compounds (OSMAC) and untargeted metabolomics approaches to investigate the bioactivities and the chemical space of the endophytic fungus *Alternaria alternata* P02PL2, isolated from a medicinal plant, *Sclerocarya birrea*. The antibacterial activities were assessed against contemporary clinical isolates, MDR *Pseudomonas aeruginosa* 5625574, methicillin-resistant *Staphylococcus aureus* (MRSA) 5627679, and a collection culture of *S. aureus* ATCC 25923 for reference. We first tested for bioactivities across eight growth media to optimize the production of bioactive secondary metabolites. The bioactive fungal extract fractions exhibited minimum inhibitory concentrations (MICs) ranging from 0.25 to 0.5 mg/mL. This was followed by an untargeted metabolomics analysis, which identified 41 secondary metabolites. Virtual screening, molecular docking, and 200-ns molecular dynamics simulations targeting *S. aureus* and *P. aeruginosa* DNA gyrase revealed lead compounds with superior binding affinities (e.g., fluorescein −9.2 kcal/mol for *P. aeruginosa*; pyrrolo[1,2-a] pyrazine-1,4-dione, hexahydro −8.8 kcal/mol for *S. aureus*) compared to reference ligands. Binding free energy calculations highlighted strong affinities, with 5,7-dihydroxy-2-(4-hydroxyphenyl)-6,8-bis(3,4,5-trihydroxyoxan-2-yl)chromen-4-one (−42.16 kcal/mol) and fluorescein (−41.42 kcal/mol) outperforming the *P. aeruginosa* reference ligand evo (−19.80 kcal/mol) and 80s (−48.74 kcal/mol) leading for *S. aureus*. Per-residue energy decomposition and quantum chemical analyses further elucidated key interactions and reactivity profiles. These results position *A. alternata* P02PL2 as a promising source of DNA gyrase inhibitors to combat antimicrobial resistance.

**Key points:**

*•* *A. alternata extracts inhibits S. aureus and Pseudomonas with MIC of 0.25-0.5 mg/mL*

*•* *Inhibitors included Diketopiperazine (S. aureus), C-glycosyl flavone (Pseudomonas)*

*•* *In silico analyses suggest the metabolites putatively inhibit bacterial DNA gyrase*

**Graphical abstract:**

**Figure:**
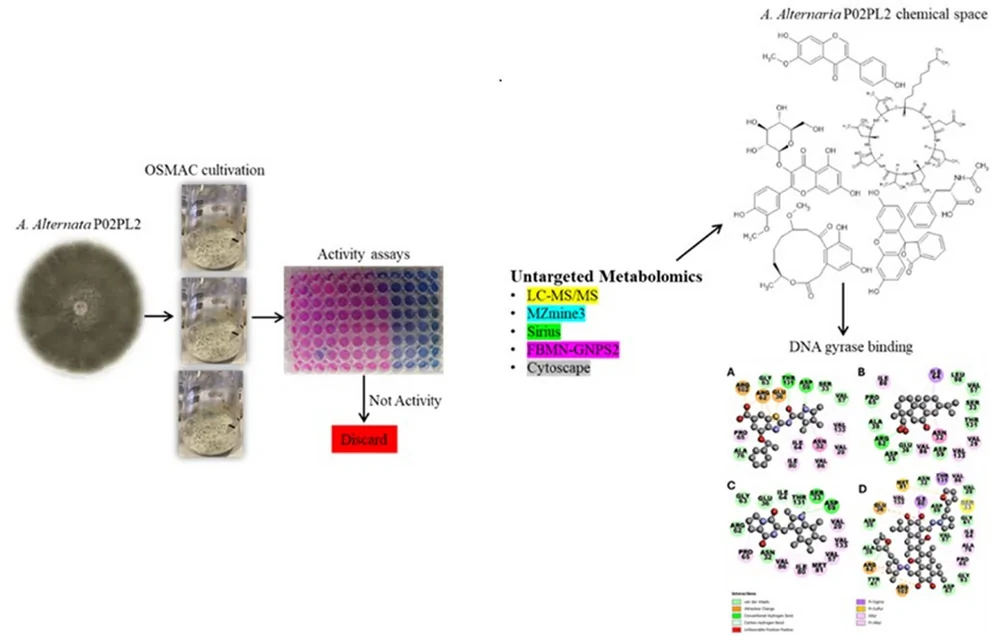

## Introduction

Antimicrobial resistance (AMR) remains a significant threat to human health, with approximately 4.95 million deaths associated with AMR annually (WHO 2022). The World Health Organization (WHO) anticipates that this value might increase to 10 million by 2050, thus underlining it as a major public health risk (Mancuso et al. 2021). In 2019, 32 antibacterial agents in clinical development that target the WHO-listed bacterial priority pathogens were identified, of which only six were classified as innovative, and none of these targets gram-negative bacteria (WHO 2019). As a result, there is a renewed interest in microbial-derived drugs to manage or cure illnesses brought on by these pathogens (Moradali et al. 2017; Tong et al. 2015). Microbial secondary metabolites are characterized by their structural diversity, rigidity, and a chemical space that spans a wide spectrum of activities, which places them as good candidates to match for mitigating the rising antimicrobial resistance challenge (Fanele and Ndlovu 2023; Macheleidt et al. 2016). To avoid rediscovering already known molecules that have masked previous phenotypic screening efforts, biodiversity mining approaches should prioritize less explored taxa, such as the microbiomes associated with plants or animals (Miethke et al. 2021). Increasing research efforts are placed on endophytic microorganisms, such as fungi, which colonize the intracellular and intercellular regions of healthy plant tissues without damaging the host plant (Nair and Padmavathy 2014; Rodriguez et al. 2009). Bioprospecting efforts are informed by ethnobotanical and traditional knowledge, which provide a rational basis for prioritizing plant species with documented pharmacological use (Buenz et al. 2004).

The secondary metabolites (SMs) are encoded by biogenetic gene clusters (BGCs), which are only expressed under certain signalling conditions, such as when they are defending the plant host from pathogens (Deepika et al. 2016; Hardoim et al. 2015). Hence, numerous fungal BGCs are silent or transiently expressed when fungi are cultivated under normal laboratory settings, and the signalling cascades that lead to their expression in the natural environment remain unknown (Pillay et al. 2022; Scherlach and Hertweck 2009; Wasil et al. 2013). Different studies have shown that small changes in cultivation conditions and medium composition (OSMAC approach) trigger the expression of silenced BGCs, thus shifting the metabolic profile of various filamentous fungi (Bode et al. 2002; González-Menéndez et al. 2019; Romano et al. 2018; Toghueo et al. 2020). This approach underlines how a single strain can produce different molecules when grown under different environmental conditions (Romano et al. 2018). Moreover, the OSMAC approach triggers a global expression of BGCs, resulting in a large chemical space. An untargeted metabolomics approach can effectively identify all detectable small metabolites in complex biological samples with enormous chemical space (Aderemi et al. 2021; Nguyen et al. 2020). This approach starts with a mass spectrometry–based analysis of biological samples, conversion of raw spectral data into a putative metabolic feature table using pre-processing tools like MZMine, followed by annotation of the chemical space by matching against spectral libraries using platforms such as the Global Natural Product Social Molecular Networking (GNPS-MN), or in silico annotation by structural prediction using programs such as Sirius (Aron et al. 2020; Shah et al. 2025). This is typically followed by statistical analysis and visualization of the chemical space as a molecular network on Cytoscape (Aron et al. 2020; Nothias et al. 2020).

Complementing these experimental approaches with computational methods such as virtual screening has become invaluable for accelerating the prediction of mechanisms of action and prioritization of antibacterial candidates. In this work, a structured virtual screening campaign was conducted targeting the DNA gyrase enzymes of *Staphylococcus aureus* and *Pseudomonas aeruginosa*, key bacterial pathogens with critical roles in antibiotic resistance. The study employed rigorous molecular docking validated by low RMSD re-docking of reference ligands, enabling the identification of lead compounds with superior predicted binding affinities compared to known inhibitors. Subsequent quantum chemical analyses, molecular dynamics simulations, and binding free energy calculations provided insights into the stability, dynamics, and energetic favorability of the protein-ligand complexes. This integrative computational framework not only prioritized compounds with favorable drug-like properties but also elucidated the molecular interactions underpinning their selective inhibition, thereby offering promising scaffolds for the development of novel antibacterial agents.

## Materials and methods

### Fungal material

The endophytic filamentous fungus, *Alternaria alternata* P02PL2, was isolated from *Sclerocarya birrea* plant (deposited at the University of KwaZulu Natal Herbarium, voucher number 18234) as previously reported in our research group (Fanele and Ndlovu 2023). The fungal isolate, *A. alternata* P02PL2 ITS sequence region of the rDNA, was deposited in the National Centre for Biotechnology Information (NCBI) under accession number OP630378 (Fanele and Ndlovu 2023). In this study, the glycerol cultures prepared from single spore cultures of *A. alternata* P02PL2 preserved in 15% glycerol and kept at −80 °C were revived by growing them on potato dextrose agar (PDA, Neogen corporation, UK) supplemented with 50 µg/mL chloramphenicol (Sigma-Aldrich, Israel) to prevent bacterial growth and cultured at 25 °C for 5 days under static conditions.

### Test microorganisms

The bacteria used as indicator organisms in this study were donated by the National Health Laboratory Services (NHLS) at Inkosi Albert Luthuli Central Hospital in Durban, South Africa. These test bacterial isolates include the gram-positive *Staphylococcus aureus* ATCC 25923, gram-positive methicillin-resistant *Staphylococcus aureus* (MRSA) 5627679, and gram-negative multidrug-resistant (MDR) *Pseudomonas* 5625574.

### Production of secondary metabolites

A fungal preculture was prepared by adding 5 mm^2^ of fungal mycelial plug from a 5-day-old fungal culture in 10 mL of pre-sterilized YM broth (3 g/L yeast extract, 3 g/L malt extract, 5 g/L peptone, 10 g/L glucose) in a 25-mL McCartney bottle followed by cultivation for 7 days at 25 °C with shaking at 150 rpm in a shaking incubator (FMH Instruments, South Africa). The production of secondary metabolites was conducted using a one-strain many compounds (OSMAC) approach, where the growth medium was systematically altered to induce the synthesis of various secondary metabolites. The fungal endophyte was subjected to solid medium fermentation by inoculating 1 mL of the YM fungal preculture into 250-mL Erlenmeyer flasks solid media: rice media (10 g in 25 mL of tap water), oatmeal media (10 g in 25 mL of tap water), and mixed media (5 g of rice and 5 g of oatmeal in 25 mL of tap water) in the dark for 14 days at 25 °C. For liquid medium fermentations, a 5-mm^2^ fungal mycelial plug from a 5-day-old fungal culture was added to 10 mL of liquid media in 25-mL McCartney bottles. The liquid media used were 1% malt extract broth (ME, Neogen corporation, USA), potato dextrose broth (PDB, Neogen corporation, USA), yeast peptone soluble starch (YPSS) (4 g/L yeast extract, 14 g/L soluble starch, 1 g/L dibasic K_2_HPO_4_, 0.5 g MgSO_4_·7H_2_O), yeast extract soy peptone dextrose (YESD) (20 g/L soy peptone, 20 g dextrose, 5 g/L yeast extract), and peptone yeast glucose (PYG) (1.25 g/L peptone, 1.25 g/L yeast extract, 5 g/L glucose). The cultures were cultivated for 14 days at 25 °C with shaking at 100 rpm in a shaking incubator (FMH Instruments, South Africa). The experimental controls consisted of each respective medium without the fungal inoculum, and the assays were performed in triplicate.

After 14 days of cultivation, fungal crude extraction was carried out by adding an equal volume of 100% ethyl acetate (ACE chemicals, South Africa) to the cultures. The samples were shaken overnight at 100 rpm at 25 °C in a shaking incubator (FMH Instruments, South Africa). The extracts were then filtered to remove mycelium using a Miracloth (Merck, USA) before being dried using a rotary evaporator (Labtech, Kempton Park, South Africa). The dried crude extracts were tested for antibacterial activity using the well diffusion assay.

### Bioactivity screening using well diffusion assays

The agar well diffusion assay was used to assess the bioactivity of the crude extracts against three indicator bacteria (*Staphylococcus aureus* ATCC 25923, methicillin-resistant *Staphylococcus aureus* (MRSA) 5627679, and multi-drug resistant (MDR) *Pseudomonas* 5625574) following a protocol described by Magaldi et al. (2004) with some modifications. The indicator bacteria were cultured on Mueller-Hinton agar (MH agar, Merck, Germany) plates at 37 °C for 12 h before being suspended in sterile distilled water and standardized against a 0.5 McFarland standard (1.5 × 10^8^ colony-forming units (CFU/mL)). A 100-µL aliquot of the respective standardized test bacterial cultures was spread evenly over the surface of the MH agar plates using a sterile cotton swab, and the plates were left to dry for 5 min. A sterile cork borer was used to drill 6-mm wells into each MH agar plate. The fungal extracts were diluted to 100 mg/mL in distilled water. Then, 80-µL aliquots of 100 mg/mL of the fungal extracts were loaded into the wells and allowed to diffuse for 10 min before the plates were incubated for 16 h at 37 °C. Following this, the zones of inhibition were observed and measured to determine the bioactivity of each fungal extract. Trimethoprim (Merck, South Africa) was used as a positive control against *S. aureus* ATCC 25923 and MRSA 5627679. Amikacin (Merck, South Africa) was used as the positive control against MDR *Pseudomonas* 5625574. The negative control were fungal-free media extracted in the same way as the experimental cultures. The assays were done in triplicate, and the endophytic fungal extracts that exhibited antibacterial activity against the test bacteria were selected for large-scale fermentation.

### Large-scale fermentation to produce secondary metabolites

#### Solid-phase extraction (SPE)

Crude extracts from rice media and oatmeal media showed inhibition against *S. aureus* ATCC 25923. On the other hand, crude extracts obtained from 1% malt extract broth, YPSS, rice, and oatmeal exhibited inhibition against MRSA 5627679. Furthermore, the fungal crude extract from potato dextrose broth exhibited inhibitory activity against the gram-negative bacterial strain MDR *Pseudomonas* 5625574. Fungal crude extracts that exhibited activity against the test organisms were fractionated using a Carbon 18 (C18, Merck, South Africa) cartridge, as detailed by Cutignano et al. (2015) or mixed-mode cartridges as described by Stoszko et al. (2020) with modifications as described by Nzimande et al. (2022).

A method described by Fanele and Ndlovu (2023) was used for solid-phase extraction (SPE) using C18 cartridges. Briefly, the C18 sorbent cartridge was conditioned with 3 mL of 100% HPLC-grade methanol (Merck, Germany). The cartridge was then equilibrated with 3 mL of sterile distilled water. After the equilibration step, 3 mL of the crude extract was loaded into the C18 cartridge. The sample was then slowly passed under a vacuum for not more than 20 pgs. This was then followed by a series of elution steps: fraction A, sterile distilled water (H_2_O); fraction B, methanol-water (CH_3_OH/H_2_O) (50:50 *v/v*); fraction C, acetonitrile-water (CH_3_CN/H_2_O) (70:30 *v/v*); fraction D, 100% acetonitrile (CH_3_CN); and fraction E, dichloromethane-acetonitrile (CH_2_CL_2_/CH_3_OH) (90:10 *v/v*). All fractions were then collected into their respective elution tubes. All the eluted fractions were concentrated by evaporation at 40 °C. The dried fractions were then reconstituted to 5 mg/mL in HPLC-grade methanol (Merck, South Africa) and stored at 4 °C until further use.

The crude extracts were also fractionated using mixed-mode cartridges: strong Anion-eXchange (MAX) and Mixed-mode strong Cation-eXchange (MCX) cartridges (Oasis, Ireland), obtained from Waters Corporation. Briefly, the MAX and MCX were conditioned with 1 mL of 100% HPLC-grade methanol (Merck, South Africa). The cartridges were then equilibrated with 1 mL of sterile distilled water. Following the equilibration step, 1 mL of the crude extract was spiked with 20 µL of 100% formic acid (Merck, India) and loaded onto MAX and MCX cartridges. The samples were then slowly passed under a vacuum for not more than 20 pgs. This was followed by stepwise elution with increasing concentration of methanol: fraction F, 5% MeOH (MAX); fraction G, 45% MeOH (MAX); fraction H, 95% MeOH (MAX); fraction I, 5% MeOH (MCX); fraction J, 45% MeOH (MCX); and fraction K, 95% MeOH (MCX). All the eluted fractions were concentrated by evaporation at 40 °C. The dried fractions were then reconstituted to 5 mg/mL in HPLC-grade methanol (Merck, South Africa) and stored at 4 °C until further use.

#### Minimum inhibitory concentrations (MICs)

The bioactivity profile of all the fractions was assessed by determining their minimum inhibitory concentrations (MICs) as detailed by Fanele and Ndlovu (2023) with minor modifications. Briefly, 50 µL of Mueller-Hinton broth (MHB, Merck, Germany) was added to a 96-well flat-bottom microtiter plate, with column 12 serving as the sterility control. After that, 100 µL of the respective fractions was added to the first column. This was followed by a twofold serial dilution from column 1 to column 11. Trimethoprim (0.65 mg/mL, Merck, Germany) was used as a positive control against *S. aureus* ATCC 25923 and MRSA 5627679, while amikacin (7.60 mg/mL, Merck, Germany) was used as the positive control against MDR *Pseudomonas* 5625574. The extract from fungi-free media was used as the negative control. An inoculum was prepared by using each bacterium from a 12-h culture on MH agar (Merck, Germany) in sterile phosphate-buffered saline (PBS, Invitrogen, USA) and standardized against a 0.5 McFarland standard (1.5 × 10^8^ colony-forming units (CFU/mL)). Then, 50 µL of the standardized inoculum was loaded into all the wells of the 96-well plate. The plates were sealed and placed in an incubator at 37 °C for 24 h. All antimicrobial activity assays were performed in triplicate, and the final concentration ranged from 0.5 to 0.00048 mg/mL. After incubation, 20 µL of 0.02% resazurin sodium salt solution (Merck, Germany) was added to each well of the plates. The plates were covered and incubated for 2 h. Bacterial inhibition was indicated by no color change of resazurin salt (remains blue), and the color change from blue to pink after incubation indicated no bacterial inhibition. The fractions that yielded positive results were filtered with a 0.22-µm filter before analysis.

#### High-resolution liquid chromatography-mass spectrometry analysis

The fungal extracts were analyzed using a Nexera LC-40D XR liquid chromatography system coupled to a quadrupole time-of-flight tandem mass spectrometer (LCMS-9030 qTOF, Shimadzu Corporation, Kyoto, Japan) following a method described by Moloi et al. (2025) with some modifications. Pooled samples representing all the injected extracts were used as quality controls to condition the LC-MS methods. Briefly, the separation was performed using a 2.7-µm particle size Shim-pack Velox C18 column (150 mm, 2.1 mm, Shimadzu Corporation, Kyoto, Japan) at 40 °C. All samples were injected at a volume of 20 μL with solvent A (0.1% formic acid, Merck, Darmstadt, Germany) and solvent B (acetonitrile:0.1% formic acid, LC-MS grade, Merck, Darmstadt, Germany). The flow rate was maintained at 0.4 mL/min throughout the 30-min gradient. The chromatographic gradient was set as follows: linear gradient of 5–65% B over 20 min, the gradient was ramped to 90% B in 3 min (20–23 min) and held at that composition for 3 min (23–26 min), the composition was returned to 5% B at 26.1 min until 30 min to recondition the column.

The Q-ToF mass spectrometer was operated in data-dependent acquisition (DDA) mode using positive electrospray ionization with the following parameters: interface voltage of 4.0 kV, interface temperature of 300 °C, nebulization, dry gas flow of 3 L/min, heat block temperature of 400 °C, DL temperature of 280 °C, detector voltage of 1.8 kV, and a flight temperature of 42 °C. The spectra, MS1 and MS2, were generated for all ions with an m/z range of 100 to 1200, exceeding an intensity threshold of 3000. Fragmentation of ions was performed using argon as a collision gas at a collision energy of 30 ± 17 eV.

#### Data processing and GNPS-molecular networking

The data obtained from the mass spectrometry was converted to an open format, mzXML file using MSconvert software (http://proteowizard.sourceforge.net/downloads.shtml; accessed 10 October 2024). The mass spectrometry data files were first processed using MZmine 3 (Schmid et al. 2023) and uploaded to the GNPS2 MN (Global Natural Products Social-Molecular Network, Version 28.2) platform (https://gnps2.org/), accessed on May 28, 2025. The data were processed using the feature-based molecular networking (FBMN) workflow online (https://ccms-ucsd.github.io/GNPSDocumentation/). Briefly, the MS/MS spectra and the GNPS libraries were window-filtered by choosing only the top six fragment ions in the ± 50 Da window throughout the spectrum. Both the precursor ion mass tolerance and the MS/MS fragment ion tolerance were set to 0.02 Da. The edges were filtered to have a cosine score above 0.7 and more than six matched peaks before a network was created. The edges between two nodes were retained in the network only if each node appeared in the other’s respective top 10 most similar nodes. The maximum size of a molecular family was set to 100, and the lowest-scoring edges were removed from molecular families until the molecular family size was below this threshold (Horai et al. 2010; Moloi et al. 2025; Nothias et al. 2020; Wang et al. 2016).

Further annotation of MS/MS spectra was done using the dereplicator (Mohimani et al. 2018) and the Sirius platform, version 5.8.5, using the built-in CSI fingerID and Canopus features (Duhrkop et al. 2019; Dührkop et al. 2021, 2015). The web interface for feature-based molecular networking (https://fbmn-statsguide.gnps2.org/; accessed 10 May 2025) was used to clean up the FBMN data, statistical analysis, and integration into the Cytoscape visualization tool (Shah et al. 2025). The molecular networks were visualized using Cytoscape software, Version 3.10.2 (Shannon et al. 2003).

#### Virtual screening and docking studies

To evaluate the biological relevance of the identified compounds, molecular docking was performed against the DNA gyrase enzymes of *P. aeruginosa* (PDB code, 7PTG) and *S. aureus* (PDB code, 5CPH). Protein preparation was performed using AutoDock Tools v.1.5.6 (Morris et al. 2009)⁠, where water molecules, ions, and co-crystallized ligands were removed. Missing side chain atoms and polar hydrogen atoms were added, and Kollman charges were assigned to the protein structure to optimize it for docking. The ligand library was first prepared in 3D format with explicit hydrogens and appropriate protonation states at pH 7.4 using OpenBabel v.2.4.1 (O’Boyle et al. 2011). These structures were then processed with Meeko v.0.6.1 (Eberhardt et al. 2020) to assign rotatable bonds and convert them to PDBQT format compatible with AutoDock Vina (Trott and Olson 2010).

The docking protocol was validated by re-docking the co-crystallized ligands, with success criteria defined as a root mean square deviation (RMSD) of less than 2.0 Å from the crystallographic pose (Pagadala et al. 2017; Warren et al. 2006). The binding site was defined based on the position of the co-crystallized ligand. A two-step docking approach was employed using AutoDock Vina: initial screening was conducted with default parameters (exhaustiveness = 8) to identify promising candidates, followed by a more rigorous docking phase for the top 10% of compounds based on binding affinity scores, using refined parameters (exhaustiveness = 128, number of binding modes = 20, and energy range = 3 kcal/mol). To ensure reproducibility and adequate sampling of conformational space, each compound was docked in triplicate using different random seeds.

Docking results were ranked according to binding affinity scores, and co-crystallized compounds 80 s and evo were selected as control ligands for *P. aeruginosa* DNA gyrase (7PTG) and *S. aureus* DNA gyrase (5CPH), respectively. Additionally, the top three scoring compounds per target were selected for subsequent analysis. The ligand binding poses and protein-ligand interactions were visualized using Discovery Studio Visualizer version 20.1.0.19295 (Biovia 2024) and PyMol v.3.0 (Schrödinger 2024).

Subsequent to docking, DFT calculations were performed using Gaussian 16 with the B3LYP/Def2TZVP level of theory (Zhao et al. 2023)⁠. This step refined the geometries of the top-scoring compounds and calculated key quantum descriptors, including HOMO-LUMO gaps, chemical potential, and electrophilicity indices. The molecular geometries of the ligands were optimized to their nearest local energy minima. To ensure that these optimized structures represented true minima, vibrational frequency analyses were conducted. The electronic characteristics of the compounds were examined by calculating the energies of the highest occupied molecular orbital (HOMO) and the lowest unoccupied molecular orbital (LUMO), along with the corresponding energy gap (∆*E*). Furthermore, global reactivity parameters such as chemical potential (*μ*), chemical hardness (*η*), chemical softness (*ζ*), electrophilicity index (*ω*), and electronegativity (*χ*) were computed based on the HOMO and LUMO energies using standard equations.

1 $$\mu=\frac{E_{HOMO}+E_{LUMO}}2$$

2 $$\eta =\frac{{E}_{LUMO}-{E}_{HOMO}}{2}$$

3 $$\zeta=\frac1\eta$$

4 $$\chi = - \mu$$

 

5 $$\omega=\frac{\mu^2}{2\eta}$$

#### Molecular dynamics simulations

Molecular dynamics (MD) simulations were performed to assess the stability and dynamic properties of protein-ligand complexes using the GPU-accelerated PMEMD module in AMBER20 (Case 2023). The protein was modelled with the ff14SB force field (Maier et al. 2015), and ligands were parameterized using the General AMBER Force Field 2 (GAFF2) (Wang et al. 2004). Ligand partial atomic charges were derived using the AM1-BCC approach within Antechamber. The systems were constructed in Tleap, solvating each complex in a TIP3P water box with a 10-Å padding and neutralizing with Na⁺ or Cl⁻ ions as needed. The SHAKE algorithm (Ryckaert et al. 1977) constrained bonds involving hydrogen atoms, long-range electrostatic interactions were handled via the Particle Mesh Ewald method (Darden et al. 1993), and a 10-Å cutoff was used for non-bonded interactions.

Energy minimization proceeded in two phases: (i) minimization of solvent and ions with the solute restrained, followed by (ii) full system minimization without restraints. The systems were heated incrementally from 0 to 300 K over 100 ps under constant volume conditions using a Langevin thermostat, followed by a 500-ps equilibration under NPT conditions at 1 bar with a Berendsen barostat (Berendsen et al. 1984). Production MD runs were conducted for 200 ns with a 2-fs time step in the NPT ensemble.

#### Post-molecular dynamics simulation analysis

Post-MD analyses were conducted using the PTRAJ and CPPTRAJ tools in AMBER (Roe & Cheatham 2013) to evaluate root mean square deviation (RMSD), root mean square fluctuations (RMSF), radius of gyration (RoG), hydrogen bond (HB) interactions, and key protein-ligand contacts. Binding free energies were calculated using the molecular mechanics/generalized born surface area (MM/GBSA) method via MMPBSA.py.MPI in AMBER20 (Miller et al. 2012). The binding energy (Δ*G*_bind_) was determined using the following relationships:

6 $$\Delta G_{bind}\:=\:G_{\mathrm{complex}}-G_{\mathrm{receptor}}-G_{\mathrm{ligand}}$$

7 $$\Delta G_{\mathrm{bind}}\:=\:E_{\mathrm{gas}}\:+\:G_{\mathrm{sol}}\;-\;T\Delta S$$

8 $$E_{gas}\:=\:E_{int}\:+\:E_{vdW}\:+\:E_{ele}$$

9 $$G_{\mathrm{sol}}\:=\:G_{\mathrm{GB}}\:+\:G_{\mathrm{SA}}$$

In these expressions, Δ*G*_bind_ represents the overall free energy of binding, *E*_gas_ corresponds to the gas-phase interaction energy, and *G*_sol_ is the solvation energy. The terms *T* and Δ*S* stand for temperature and entropy changes of the system, respectively. *G*_GB_ and *G*_SA_ account for polar and non-polar contributions to solvation energy, while *E*_vdW_ and *E*_ele_ reflect van der Waals and electrostatic components. Additionally, a per-residue energy decomposition analysis was conducted to identify key residues that significantly contribute to ligand binding affinity.

## Results

### Cultivation and bioactivity screening of crude extracts from A. alternata P02PL2

The fungal endophyte used in this study, *A. alternata* P02PL2, was first reported by Fanele and Ndlovu (2023) in our group. Crude extracts of *A. alternata *P02PL2 were produced from various media, including rice media, oatmeal media, mixed rice and oats (RO) media, 1% malt extract broth, potato dextrose broth, YPSS broth, YESD broth, and PYG broth. The resulting extracts were assessed for their bioactivity against three different test organisms, *S. aureus* ATCC 25923, MRSA 5627679, and MDR *Pseudomonas* 5625574 (Table S1). Active fungal crude extracts obtained from oatmeal media were selected for large-scale secondary metabolite production due to their potent antibacterial activity against *S. aureus* ATCC 25923 and MRSA 5627679. In addition, fungal crude extracts obtained from potato dextrose broth were also selected for large-scale secondary metabolite production because they are the only fungal crude extracts that showed repeatable activity against MDR *Pseudomonas* 562554.

### Minimum inhibitory concentrations (MICs) of crude extract fractions

Solid-phase extraction (SPE) was performed to fractionate the large-scale fungal crude extracts obtained from oatmeal media and potato dextrose broth media using C18 sorbent, MCX, and MAX cartridges. Solid-phase extraction yielded a total of 15 fractions, comprising seven from the C18 cartridge and four from the MCX and MAX cartridges. All the fractions were tested for bioactivity against three indicator strains, *S. aureus* ATCC 25923, MRSA 5627679, and MDR *Pseudomonas* 5625574. No activity was observed against MRSA 5627679 (Tables S2–S5) for any of the tested fractions. Three fractions exhibited activity against MDR *Pseudomonas* 5625574 (FF, FJ, and FK; Table 1), and two fractions exhibited activity against *S. aureus* ATCC 25923 (FD, FH; Table 1). The fungi-free crude extracts served as the negative control. Antibiotic trimethoprim was the positive control against *S. aureus* ATCC 25923, and antibiotic amikacin was the positive control against MDR *Pseudomonas* 5625574.

**Table 1:** Minimum inhibitory concentrations of *Alternaria alternata* P02PL2 fractions

Bacterial strain	Minimum inhibitory concentration (mg/mL)						
	C18 fraction D	MAX fraction F	MAX fraction H	MCX fraction J	MCX fraction K	Negative control	Positive control
MDR *Pseudomonas* 5625574	*	0.5	*	0.5	0.25	-	3.80
*S. aureus* ATCC 25923	0.5	*	0.25	*	*	-	0.65

*C18* carbon 18, *MAX* mixed-mode strong anion-exchange, *MCX* mixed-mode strong cation-exchange, *ATCC* American Type Culture Collection, *MDR* multidrug-resistant, - no activity, * not tested against the bacterial strain

### Feature-based molecular network analysis

**Fig. 1:**
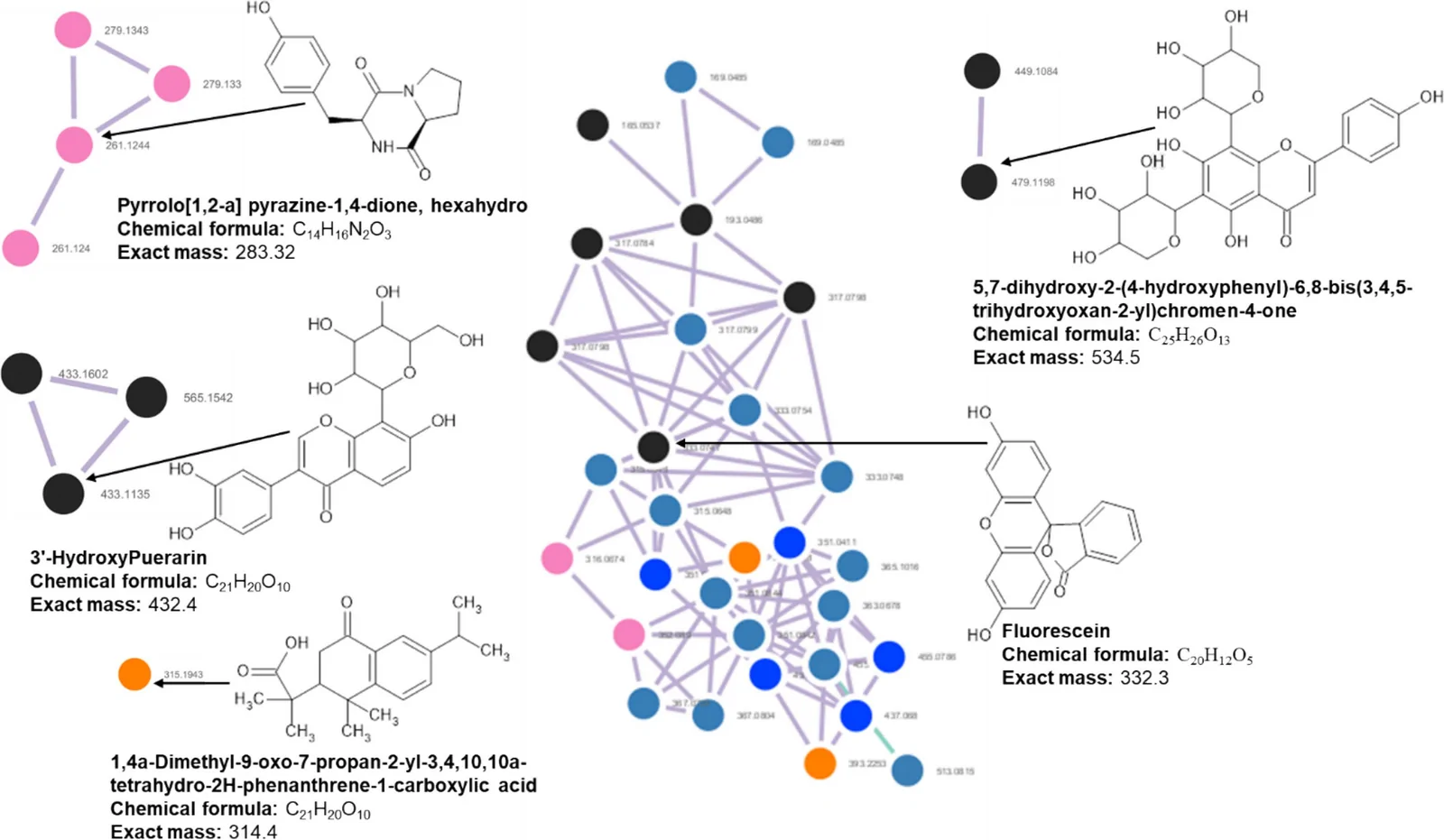
Feature-based molecular network of *Alternaria alternata* P02PL2 clusters containing molecules showing high binding affinity to DNA gyrase

The active fractions are respectively referred to as fraction K (FK; 95% MeOH, MCX), fraction F (FF; 5% MeOH, MAX), fraction D (FD; 100% acetonitrile, C18), and fraction H (FH; 95% MeOH, MAX) throughout this manuscript. All these fractions were subjected to HPLC-MS analysis. A total of 46 secondary metabolites were detected in all four fractions and confirmed by searching against the Coconut Natural Products database (Chandrasekhar et al., 2024). After carefully scanning the features of the metabolites, only 39 secondary metabolites (Table S6) were identified as having significant intensities. The MS1 and MS2 spectra of all the identified compounds are shown in Figures S1 and S2. A feature-based molecular network (FBMN) was constructed to include selected compounds showing the highest affinity to DNA gyrase (Fig. 1).

### Virtual screening studies

Molecular docking methodology was rigorously validated by re-docking the native co-crystallized reference ligands. For *P. aeruginosa* DNA gyrase, the reference ligand evo displayed excellent docking precision with an RMSD of 0.31 Å, while the *S. aureus* DNA gyrase reference ligand 80 s showed strong accuracy with an RMSD of 0.83 Å (Figure S3). Both values fall within the acceptable threshold of < 2.0 Å, supporting the reliability of subsequent docking, molecular dynamics, and binding energy analyses with virtual screening hits.

**Fig. 2:**
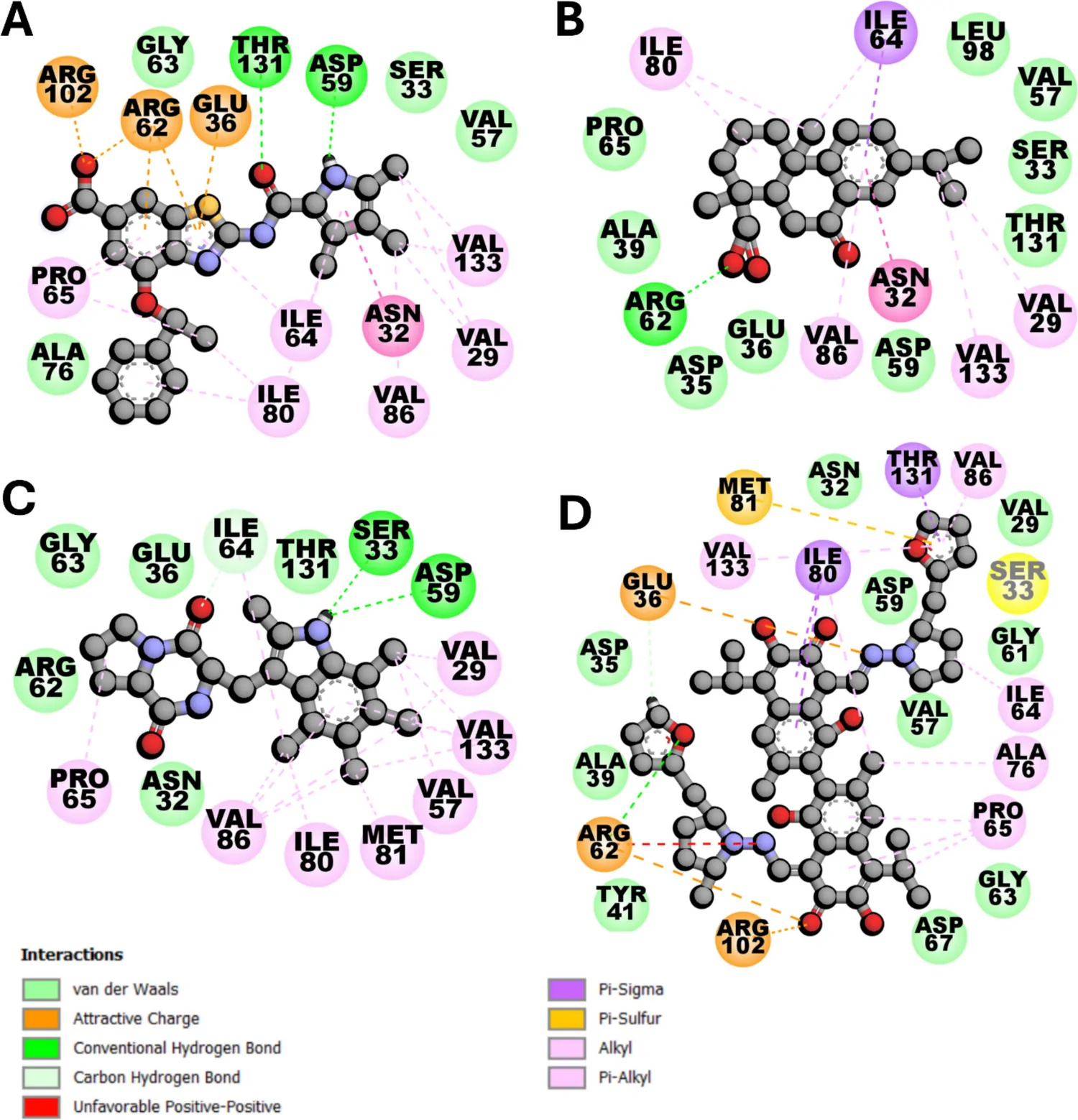
Molecular docking analysis showing key binding interactions and residue contacts for **A** 80 s, **B** CID 10018535, **C** CID 434562, and **D** CID 4643973 within the *S. aureus* DNA gyrase active site

The virtual screening campaign identified multiple promising compounds with binding affinities superior to the reference ligands (Table 2, Fig. 2). For *S. aureus*, pyrrolo[1,2-a] pyrazine-1,4-dione, hexahydro (CID 434562) showed the highest affinity (−8.8 kcal/mol), followed by 1,4a-dimethyl-9-oxo-7-propan-2-yl-3,4,10,10a-tetrahydro-2H-phenanthrene-1-carboxylic acid (CID 10018535) and CID 4643973 (both −8.7 kcal/mol), all outperforming the reference ligand 80 s (−8.1 kcal/mol). For *P. aeruginosa*, fluorescein (CID 16850) demonstrated the strongest binding energy (−9.2 kcal/mol), with 5,7-dihydroxy-2-(4-hydroxyphenyl)−6,8-bis(3,4,5-trihydroxyoxan-2-yl)chromen-4-one (CID 14427338, −9.1 kcal/mol) and CID 74218732 (−8.9 kcal/mol) also surpassing the reference ligand evo (−7.4 kcal/mol).

**Table 2:** Molecular docking results and physicochemical properties of lead compounds against *S. aureus* and *P. aeruginosa* DNA gyrase

Compound	BA (kcal/mol)	MW (Da)	LogP	HBD	HBA	TPSA (Å^2^)	RotB
*S. aureus*							
CID 434562	− 8.8	283	1.2	2	2	65	2
CID 80s	− 8.1	222	2.6	1	2	42	1
CID 10018535	− 8.7	314	4.5	1	3	54	6
CID 4643973	− 8.7	829	10.4	6	12	179	24
*P. aeruginosa*							
CID 74218732	− 8.9	695	1.1	5	13	191	16
CID evo	− 7.4	222	3.2	1	3	45	1
CID 14427338	− 9.1	534	−1.1	9	13	231	12
CID 16850	− 9.2	332	3.7	2	5	76	2

Physicochemical evaluation revealed that some lead compounds maintain favorable drug-like properties. CID 434562 and CID 10018535 for *S. aureus* targets conform well to Lipinski’s rule of five, with molecular weights of 283 Da and 314 Da and acceptable LogP values (1.2 and 4.5). By contrast, CID 4643973 violates multiple drug-likeness criteria, including excessive molecular weight (829 Da), LogP (10.4), and TPSA (179 Å^2^), suggesting limited pharmaceutical potential. Similarly, CID 16850 for *P. aeruginosa* exhibits optimal properties (MW 332 Da, LogP 3.7), while CID 74218732 and CID 14427338 show suboptimal features due to high molecular weights and polar surface areas.

Molecular docking analyses detailed distinct binding interactions within the reference ligand-compared active sites. The *S. aureus* reference ligand 80 s engaged pivotal catalytic residues via attractive charge interactions with ARG102, GLU36, and ARG62, conventional hydrogen bonds with THR131 and ASP59, and extensive van der Waals contacts with SER33, ASP59, VAL133, VAL29, and ILE64 (Fig. 2A). CID 10018535, CID 434562, and CID 4643973 exhibited diverse interaction profiles involving hydrogen bonding, hydrophobic contacts, and pi-alkyl interactions, with CID 4643973 displaying the most comprehensive network (Fig. 2B–D).

**Fig. 3:**
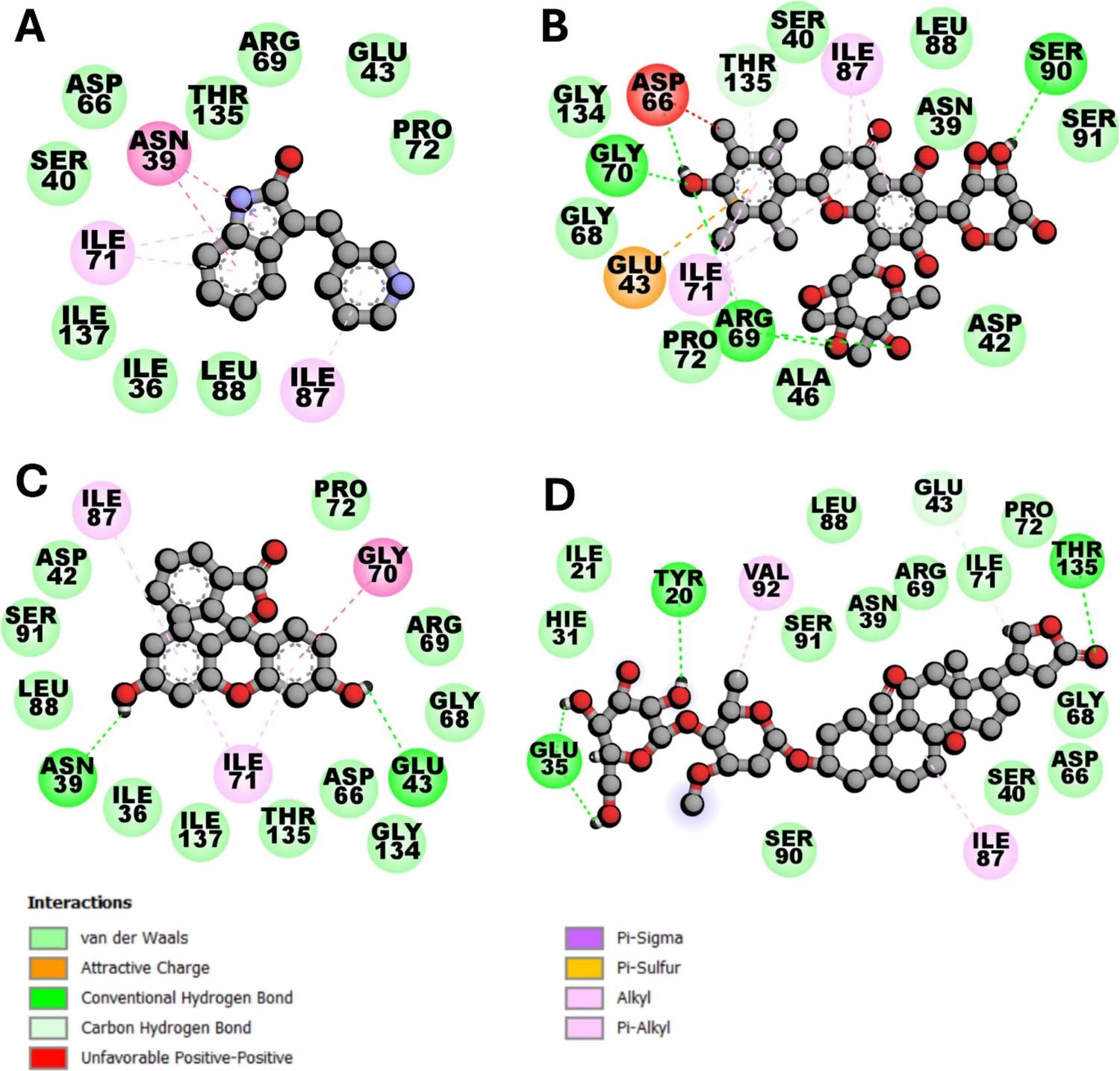
Molecular docking analysis showing key binding interactions and residue contacts for **A** evo, **B** CID 14427338, **C** CID 16850, and **E** CID 74218732 within the *P. aeruginosa* DNA gyrase active site

In *P. aeruginosa*, the reference ligand evo primarily formed van der Waals and pi-alkyl interactions with residues such as SER40, ASP66, THR135, GLY70, and ILE87 (Fig. 3A). CID 14427338 offsets charge repulsion with ASP66 via stabilizing hydrogen bonds and displays robust hydrophobic interactions (Fig. 3B). CID 16850 and CID 74218732 consistently maintained compact yet effective binding via combinations of hydrogen bonds, van der Waals forces, and pi-alkyl contacts (Fig. 3C, D).

### Quantum chemical calculations

Density functional theory (DFT) at the B3LYP/def2-TZVP level yielded stable optimized geometries for all compounds, with frequency calculations confirming the absence of imaginary modes (Figure S4). HOMO-LUMO gaps varied, with CID 4643973 showing the smallest gap (3.16 eV) among *S. aureus* hits, indicating higher reactivity, and CID 74218732 exhibiting the largest gap (5.39 eV) among *P. aeruginosa* hits, reflecting greater chemical stability. Chemical hardness and softness measures further demonstrated diversity in molecular reactivity across compounds. Notably, the *P. aeruginosa* reference ligand evo exhibited the highest electrophilic index (7.11 eV), consistent with its known biological efficacy. These quantum descriptors support the promising electronic profiles of the screened compounds in selectively targeting DNA gyrase in both bacterial species (Table 3).

**Table 3:** Quantum chemical descriptors and reactivity parameters of candidate inhibitors targeting *S. aureus* and *P. aeruginosa* DNA gyrase

Compound	*E*_*H*_ (eV)	*E*_*L*_ (eV)	∆*E* (eV)	*µ* (eV)	*ɲ* (eV)	*ꭕ* (eV)	*ζ* (eV^−1^)	*Ꞷ* (eV)
*S. aureus*								
80s	− 6.54	− 2.40	4.14	− 4.47	2.07	4.47	0.48	4.83
CID 10018535	− 6.73	− 1.71	5.01	− 4.22	2.51	4.22	0.40	3.55
CID 434562	− 5.79	− 0.83	4.95	− 3.31	2.48	3.31	0.40	2.21
CID 464397	− 5.13	− 1.97	3.16	− 3.55	1.58	3.55	0.63	3.98
*P. aeruginosa*								
evo	− 5.95	− 3.08	2.87	− 4.51	1.43	4.51	0.70	7.11
CID 16850	− 6.40	− 1.70	4.70	− 4.05	2.35	4.05	0.43	3.49
CID 74218732	− 6.95	− 1.55	5.39	− 4.25	2.70	4.25	0.37	3.35
CID 14427338	− 6.27	− 2.20	4.07	− 4.24	2.03	4.24	0.49	4.41

### Molecular dynamics simulations

**Fig. 4:**
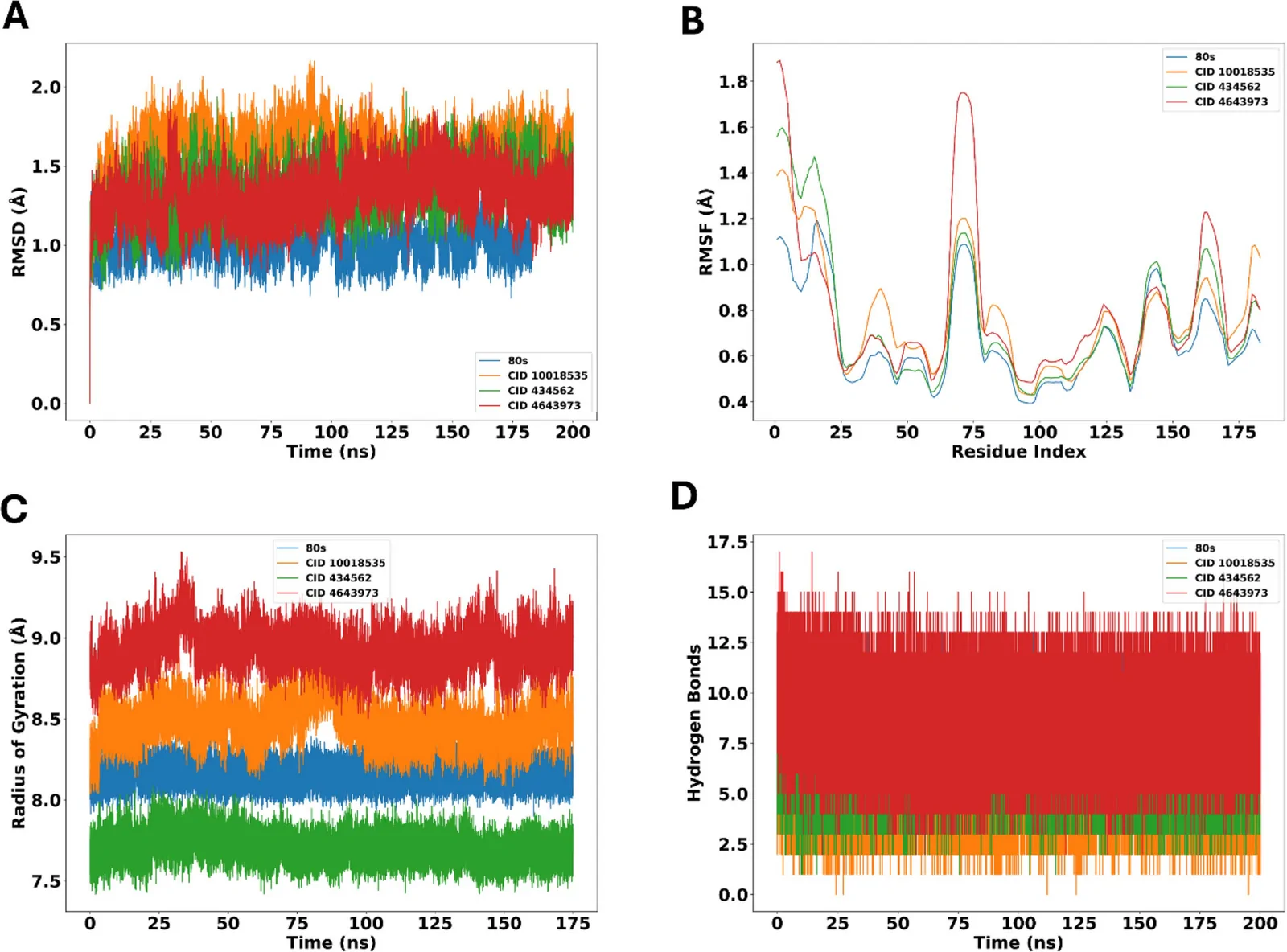
Active site dynamics during 200-ns molecular dynamics simulations of *S. aureus* DNA gyrase complexes with compounds 80 s, CID 10018535, CID 434562, and CID 4643973. **A** Root mean square deviation (RMSD) of binding site residues, **B** root mean square fluctuation (RMSF) of individual residues within the active site, **C** radius of gyration (RoG) of the ligand-binding pocket, and **D** number of intermolecular hydrogen bonds between ligand and protein

The 200-ns molecular dynamics simulations revealed stable protein-ligand complexes after equilibration (~ 50 ns). For *S. aureus*, the reference ligand 80 s maintained the most stable binding site RMSD (1.02 Å), with CID 4643973, CID 434562, and CID 10018535 showing modestly higher but stable RMSDs (Fig. 4A). RMSF analyses revealed minimal binding pocket perturbations induced by 80 s, whereas CID 10018535 caused greater residue fluctuations (Fig. 4B). Ligand RoG values were consistent across systems (7.7–8.1 Å), suggesting ligand conformational stability (Fig. 4C). Hydrogen bond analysis revealed persistent ligand-protein contacts, particularly notable for CID 4643973, which formed up to nine stable hydrogen bonds despite moderate structural flexibility (Fig. 4D).

**Fig. 5:**
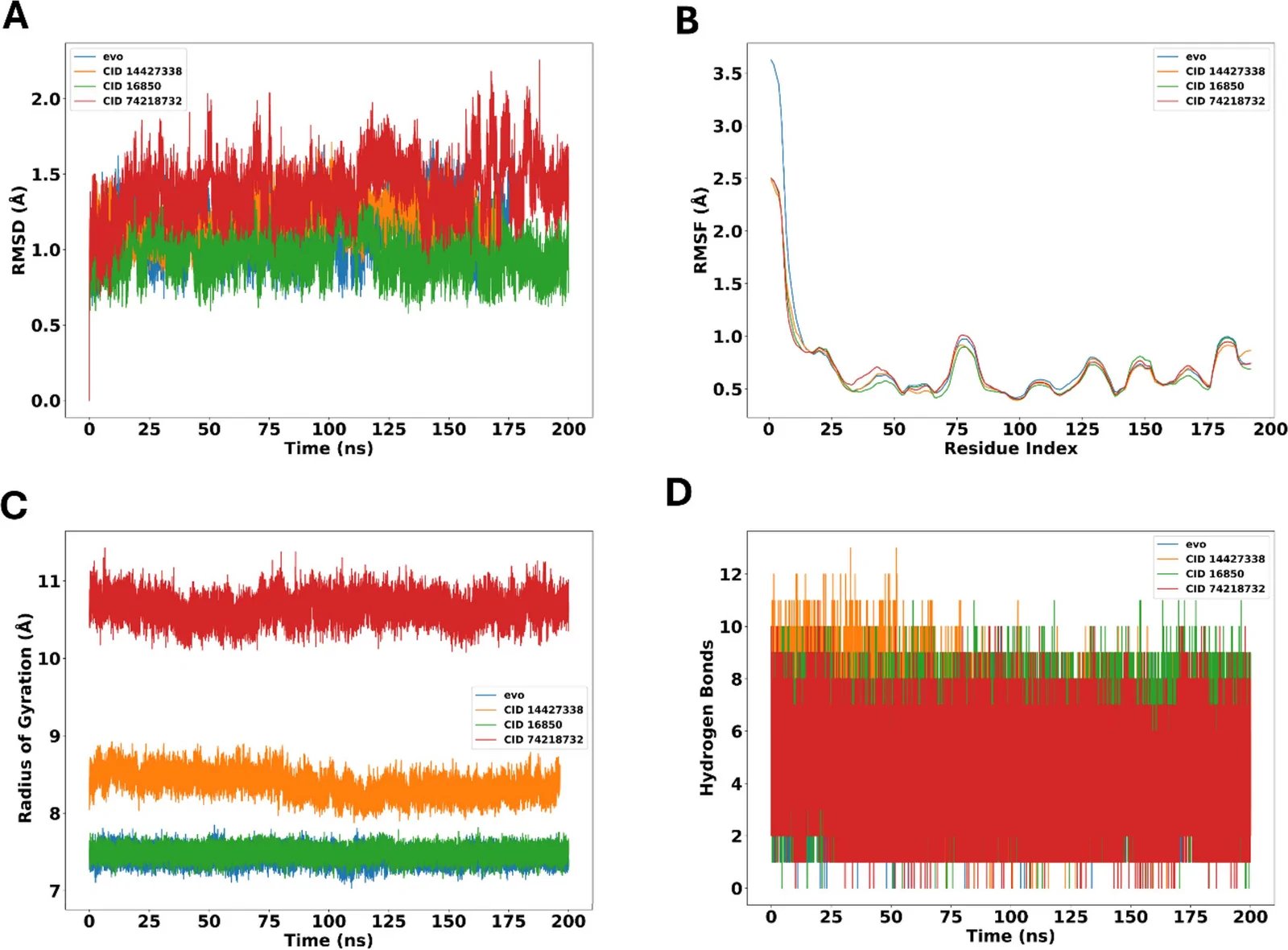
Active site dynamics during 200-ns molecular dynamics simulations of *P. aeruginosa* DNA gyrase complexes with reference compound evo and virtual screening hits CID 14427338, CID 16850, and CID 74218732. **A** Root mean square deviation (RMSD) of binding site residues, **B** root mean square fluctuation (RMSF) of individual residues within the active site, **C** radius of gyration (RoG) of ligands, and **D** number of intermolecular hydrogen bonds between ligand and protein

In *P. aeruginosa*, CID 16850 stabilized the binding pocket most effectively, achieving a lower RMSD (0.96 Å) compared to the reference ligand evo (1.14 Å) (Fig. 5A). Corresponding RMSF data indicated reduced residue mobility with CID 16850 (0.69 Å) versus evo (0.74 Å) (Fig. 5B). Ligand compactness varied with size but remained stable, with RoG spanning 7.44 Å (evo) to 10.64 Å (CID 74218732) (Fig. 5C). CID 14427338 formed the most persistent intermolecular hydrogen bonds (average 5.38), substantiating its strong binding affinity (Fig. 5D).

### Binding free energy calculations

Binding free energy calculations corroborated docking and simulation results. The *S. aureus* reference ligand 80 s exhibited the strongest binding free energy (Δ*G*_bind_ = − 48.74 kcal/mol), driven by synergistic van der Waals (− 48.15 kcal/mol) and polar solvation (− 63.94 kcal/mol) effects despite unfavorable electrostatics (+ 68.81 kcal/mol) (Table 4). Other compounds trailed with weaker affinities: CID 10018535 (− 28.74 kcal/mol), CID 4643973 (− 23.14 kcal/mol), and CID 434562 (− 20.07 kcal/mol).

**Table 4:** Binding free energy contributions of selected compounds to *S. aureus* and *P. aeruginosa* DNA gyrase targets

Compound	Δ***G***_bind_ (kcal/mol)	Δ***E***_vdw_ (kcal/mol)	Δ***E***_ele_ (kcal/mol)	Δ***G***_pol_ (kcal/mol)	Δ***G***_nonpol_ (kcal/mol)
*S. aureus*					
80s	− 48.74 ± 3.00	− 48.15 ± 3.07	68.81 ± 9.83	− 63.94 ± 8.45	− 5.45 ± 0.24
CID 10018535	− 28.74 ± 4.07	− 28.91 ± 2.92	− 10.21 ± 8.06	14.28 ± 5.70	− 3.90 ± 0.29
CID 434562	− 20.07 ± 2.86	− 27.75 ± 2.01	− 14.92 ± 5.66	25.64 ± 3.49	− 3.04 ± 0.14
CID 4643973	− 23.14 ± 3.05	− 31.38 ± 3.17	− 12.36 ± 4.53	24.36 ± 5.02	− 3.77 ± 0.35
*P. aeruginosa*					
evo	− 19.80 ± 1.96	− 25.51 ± 1.72	− 9.63 ± 2.75	18.65 ± 1.99	− 3.31 ± 0.13
CID 14427338	− 42.16 ± 5.76	− 45.05 ± 4.77	− 32.68 ± 15.50	41.95 ± 9.74	− 6.39 ± 0.31
CID 16850	− 41.42 ± 2.38	− 36.54 ± 2.67	− 44.32 ± 3.82	43.95 ± 3.15	− 4.50 ± 0.15
CID 74218732	− 30.37 ± 4.66	− 32.08 ± 4.34	− 53.74 ± 11.56	60.32 ± 10.60	− 4.87 ± 0.53

For *P. aeruginosa*, CID 14427338 (− 42.16 kcal/mol) and CID 16850 (− 41.42 kcal/mol) markedly outperformed the reference ligand evo (− 19.80 kcal/mol), with optimal balances of van der Waals and electrostatic interactions offset by polar solvation penalties. CID 74218732 exhibited a moderate affinity, consistent with the complexity of its binding profile.

Residue-level energy decomposition highlighted interaction mechanisms for ligand binding. For *S. aureus*, the reference ligand 80 s showed strong electrostatic attraction with ARG102 (−44.13 kcal/mol) balanced by solvation, and GLU36 had a net slight positive effect (+ 0.43 kcal/mol). Compounds CID 10018535 and CID 434562 exhibited moderate van der Waals interactions at ASN32 (−3.32 kcal/mol) and ARG62 (−3.78 kcal/mol), respectively, whereas CID 4643973 showed weaker overall residue contributions (Figure S5). In *P. aeruginosa*, evo engaged hydrophobic residues ARG69 (−2.60 kcal/mol) and ILE71 (−2.33 kcal/mol). CID 14427338 and CID 16850 presented strong electrostatic attraction at ASP66 (−13.15 and −12.82 kcal/mol) partially offset by solvation, with additional favorable van der Waals contacts. CID 74218732 displayed complex energetics with strong electrostatics at GLU35 (−22.19 kcal/mol) balanced by solvation (Figure S6).

## Discussion

Fungal endophytes are considered one of the most important sources of bioactive compounds with a wide range of biological activities, including antibacterial, antimicrobial, antioxidant, anticancer, antiviral, and antimalarial activities (Deshmukh et al. 2022; Khalil et al. 2021; Sharaf et al. 2022). The active compounds are encoded by biosynthetic pathways, which are classified based on the conserved backbone into polyketides, non-ribosomal peptides, terpenes, alkaloids, etc. (Ancheeva et al. 2020; Toghueo et al. 2020).

Here, we demonstrate the potential of combining a systematic cultivation approach (OSMAC), untargeted metabolomics, and in silico predictions to prioritize bioactive compounds from complex chemical space and predict their mechanism of action. The OSMAC approach has been identified as a crucial tool for the systematic and global activation of silent SM pathways in fungi (Gakuubi et al. 2021). The fungal species, *A. alternata* P02PL2, employed in this study was previously isolated from the leaves of a medicinal plant, *S. birrea*, and profiled for antibacterial activity (Fanele & Ndlovu 2023). Eight media (solid and liquid) varieties were used for preliminary bioactivity screening. Fungal crude extracts were generated and screened against three indicator organisms, including one type strain, *S. aureus* ATCC 25923, and two drug-resistant contemporary clinical isolates, MRSA 5627679 and MDR *Pseudomonas* 5625574. Only the crude extracts from rice media and oatmeal media showed inhibition against *S. aureus* ATCC 25923. On the other hand, crude extracts obtained from 1% malt extract broth, YPSS, rice, and oatmeal exhibited inhibition against MRSA 5627679. Furthermore, the fungal crude extract from potato dextrose broth exhibited inhibitory activity against MDR *Pseudomonas* 5625574. *Pseudomonas aeruginosa* is a highly resistant organism that has been listed by the WHO as one of the priority pathogens (Vo et al. 2022; WHO 2019).

Fungal crude extracts that had potent antibacterial activities were selected for large-scale production of secondary metabolite crude extracts. These fungal crude extracts were further fractionated using three SPE matrices (C18, MAX, and MCX) to simplify the complex crude extracts (Makhwitine et al. 2023). The different SPE matrices were selected based on the rationale that they can retain a wide range of organic analytes, from nonpolar to very polar analytes, from various samples (Andrade-Eiroa et al. 2016). In this study, 15 fractions were obtained from both oatmeal media and potato dextrose media using SPE. These included seven fractions from the C18 cartridge and four fractions from each of the MCX and MAX cartridges. The minimum inhibitory concentration (MIC) assays on the fractions were performed based on the preliminary activity profiles of these extracts. Therefore, fractions obtained from potato dextrose broth were evaluated against MDR *Pseudomonas* 5625574, whereas fractions obtained from oatmeal media were evaluated against *S. aureus* ATCC 25923 and MRSA 5627679. None of the tested fractions exhibited activity against MRSA 5627679 (Tables S1–S4). These findings suggest that activity against MRSA 5627679 may have been lost during the fractionation procedure. Similar findings have been reported by Nwodo et al. (2010) where they observed a decrease in antibacterial activity after fractionation of crude extracts obtained from *Tamarindus indica.* Fractions from oatmeal media (FD and FH) had MIC values ranging from 0.25 to 0.5 mg/mL, inhibiting *S. aureus* ATCC 25923. Similarly, fractions from potato dextrose broth (FF, FJ, and FK) had MIC values ranging from 0.25 to 0.5 mg/mL, inhibiting MDR *Pseudomonas* 5625574. Similar results were observed in a study conducted by Maliehe et al. (2022). The MIC values of extracts with ≤ 1 mg/mL are classified as indicative of good antibacterial activity, while extracts with MIC values > 1 mg/mL are classified as non-inhibitors (Asong et al. 2019; van Vuuren 2008).

High-resolution liquid chromatography-tandem mass spectrometry (HRLCMS/MS) analysis of the bioactive fractions resulted in the putative identification of 41 secondary metabolites. To predict the molecular mechanisms underlying the antibacterial activity of these metabolites, we conducted a virtual screening, molecular docking, molecular dynamics (MD) simulations, and binding free energy calculations targeting the DNA gyrase enzymes of *S. aureus* and *P. aeruginosa*. DNA gyrase, a critical enzyme in bacterial DNA replication, is a well-validated target for antibiotics, such as fluoroquinolones, due to its essential role in bacterial survival. Previous studies have also shown that *Alternaria* species exhibit various secondary metabolites that target both human and bacterial DNA gyrase enzymes (Jarolim et al. 2016). Our docking methodology was rigorously validated, achieving RMSD values of 0.31 Å for *P. aeruginosa* (evo) and 0.83 Å for *S. aureus* (80s), confirming high docking precision. The virtual screening campaign identified several compounds with superior binding affinities compared to the reference ligands, notably pyrrolo[1,2-a]pyrazine-1,4-dione, hexahydro (CID 434562, −8.8 kcal/mol) and 1-phenanthrenecarboxylic acid (CID 10018535, −8.7 kcal/mol) for *S. aureus* and fluorescein (CID 16850, −9.2 kcal/mol) and 5,7-dihydroxy-2-(4-hydroxyphenyl)−6,8-bis(3,4,5-trihydroxyoxan-2-yl)chromen-4-one (CID 14427338, −9.1 kcal/mol) for *P. aeruginosa*. In a study by Kiran et al. (2018), it was demonstrated that pyrrolo[1,2-a]pyrazine-1,4-dione, hexahydro, isolated from a novel *Bacillus tequilensis* MSI45 strain, exhibited antioxidant and antibacterial activity against multidrug-resistant *S. aureus* with an MIC of 15 ± 0.172 mg/L. There is no recorded antibacterial activity for the other compounds identified in this study; however, phenanthrenes (CID 10018535) and flavonoids (CID 14427338) have been shown in previous studies to exhibit antimicrobial activities against MDR *S. aureus*(Chen et al. 2018) and *Pseudomonas aeruginosa*(Zhang et al. 2025), respectively. The binding affinities of the identified compounds suggest that these compounds may be putative inhibitors of DNA gyrase, potentially disrupting bacterial DNA replication. There are no previous studies that have linked the identified compounds to DNA gyrase inhibition; these predictions require further validation using in vitro studies.

The physicochemical evaluation of these lead compounds revealed that pyrrolo[1,2-a] pyrazine-1,4-dione, hexahydro (CID 434562) and 1,4a-dimethyl-9-oxo-7-propan-2-yl-3,4,10,10a-tetrahydro-2H-phenanthrene-1-carboxylic acid (CID 10018535) for *S. aureus*, and fluorescein (CID 16850) for *P. aeruginosa* conform well to Lipinski’s rule of five, indicating favorable drug-like properties. In contrast, compounds like 2-[8-[[2-(furan-2-ylmethyl)−5-methylpyrrolidin-1-yl]iminomethyl]−1,6,7-trihydroxy-3-methyl-5-propan-2-ylnaphthalen-2-yl]−8-[[2-(furan-2-ylmethyl)pyrrolidin-1-yl]iminomethyl]−3-methyl-5-propan-2-ylnaphthalene-1,6,7-triol (CID 4643973) and apobioside (CID 74218732) exhibited suboptimal characteristics, such as excessive molecular weight and high polar surface areas, which may limit their pharmaceutical applicability. These findings highlight the importance of integrating computational screening with physicochemical assessments to prioritize candidates with optimal pharmacokinetic profiles. Quantum chemical calculations using DFT further supported the reactivity profiles of the lead compounds. Notably, CID 4643973 exhibited the smallest HOMO-LUMO gap (3.16 eV) among *S. aureus* hits, indicating higher reactivity, while CID 74218732 showed the largest gap (5.39 eV) among *P. aeruginosa* hits, suggesting greater chemical stability. The high electrophilic index of the reference ligand evo (7.11 eV) aligns with its known biological efficacy, while the favorable electronic profiles of the screened compounds suggest their potential to selectively target DNA gyrase.

Molecular dynamics simulations provided further insights into the stability and dynamics of the protein-ligand complexes. For *S. aureus*, the reference ligand 80 s exhibited the most stable binding site RMSD (1.02 Å), while CID 4643973 formed up to nine persistent hydrogen bonds, suggesting robust interactions despite its moderate structural flexibility. In *P. aeruginosa*, CID 16850 demonstrated superior binding pocket stabilization (RMSD 0.96 Å) compared to the reference ligand evo (1.14 Å), with CID 14427338 forming the most persistent hydrogen bonds (average 5.38). These results indicate that the identified compounds form stable and dynamic interactions with key residues in the DNA gyrase active sites, supporting their potential as effective inhibitors.

Binding free energy calculations corroborated the docking and MD results, with CID 14427338 (−42.16 kcal/mol) and CID 16850 (−41.42 kcal/mol) outperforming the *P. aeruginosa* reference ligand evo (−19.80 kcal/mol). For *S. aureus*, the reference ligand 80 s exhibited the strongest binding free energy (−48.74 kcal/mol), but the lead compounds showed competitive affinities. Per-residue energy decomposition analyses revealed key interactions, such as the strong electrostatic attraction of CID 14427338 and CID 16850 with ASP66 in *P. aeruginosa*, and the balanced electrostatic and van der Waals contributions of 80 s with ARG102 in *S. aureus*. These interactions underscore the molecular basis for the observed antibacterial activity and provide a framework for rational drug design.

## Conclusion

Antimicrobial resistance remains one of the most urgent global health threats, with MDR *Staphylococcus aureus* and *Pseudomonas aeruginosa* among the most challenging pathogens to treat. Here, we show that the endophytic fungus *Alternaria alternata* P0PL2, isolated from the medicinal plant *Sclerocarya birrea*, produces secondary metabolites with potent antibacterial activity, achieving minimum inhibitory concentrations as low as 0.25 mg/mL. The integration of systematic cultivation using the OSMAC approach, coupled with untargeted metabolomics, revealed a complex chemical space comprising 40 annotated metabolites. Furthermore, we demonstrated that in silico pipelines can provide a mechanistic framework for prioritizing metabolites of interest from the complex metabolite profile output of the untargeted metabolomics approach. Here, we further used in silico analysis to show that several metabolites have distinct binding affinities and putatively bind to DNA gyrase of *S. aureus* and *Pseudomonas*. Metabolites such as CIDs 16850 and 14427338 exhibited stronger in silico binding affinities for bacterial DNA gyrase than reference ligands. Molecular dynamics simulations confirmed their stability, with CID 16850 maintaining lower RMSD values than the reference ligand, and CID 14427338 forming persistent hydrogen bonds throughout the 200-ns simulation. Crucially, binding free energy analyses reinforced these findings: CID 14427338 (Δ*G*_bind_ = −42.16 kcal/mol) and CID 16850 (Δ*G*_bind_ = −41.42 kcal/mol) both displayed binding strengths more than twice as favorable as the reference ligand evo (Δ*G*_bind_ =−19.80 kcal/mol). However, the MIC values reported in this study were determined using partially purified extracts, whereas the binding free energies were calculated for individual metabolites identified through in silico analyses. Consequently, the observed discrepancy between the moderate antimicrobial activities and the strong predicted binding affinities should be interpreted with caution, as these results reflect different levels of sample complexity and experimental evaluation.

## Supplementary information

Additional data can be found in a supplementary file.

## Data Availability

The positive Feature-Based Molecular Networking data is available in the GNPS2 web interface. The LC-HRMS/MS metabolomics data have been deposited in Massive. Alternatively, all data can be accessed from the authors on request.
